# Umweltrisiken und Gesundheitskompetenz: Eine systematische Übersichtsarbeit

**DOI:** 10.1007/s00103-023-03782-5

**Published:** 2023-10-12

**Authors:** Elisabeth Pfleger, Regina Lutz, Hans Drexler

**Affiliations:** 1 Institut und Poliklinik für Arbeits‑, Sozial- und Umweltmedizin, Friedrich-Alexander-Universität Erlangen-Nürnberg, Henkestraße 9–11, 91054 Erlangen, Deutschland; 2 FOM Hochschule für Oekonomie & Management, Essen, Deutschland

**Keywords:** Gesundheitskompetenz, Umweltbezogene Gesundheitskompetenz, Umwelt, Gesundheit, Risikokommunikation, Health literacy, Environment, Health, Environmental health literacy, Risk communication

## Abstract

**Hintergrund:**

Bei der Entstehung von Erkrankungen können Umweltfaktoren eine wesentliche Rolle spielen. Das Verständnis der Beziehung zwischen Umweltrisiken und Gesundheit in der Bevölkerung gestaltet sich jedoch noch schwierig. Ziel der Arbeit ist es, den Stand der Forschung zum Zusammenhang zwischen Gesundheitskompetenz (GK) bzw. umweltbezogener Gesundheitskompetenz (UGK) und Umweltrisiken differenziert nach untersuchten Umweltrisiken, Population, Erhebung und Ausprägung der GK bzw. UGK und deren Interaktion mit anderen Variablen darzustellen.

**Methoden:**

Es erfolgte eine systematische Literaturrecherche in den Datenbanken Pubmed, Scopus und LIVIVO. Eingeschlossen wurden Originalarbeiten in deutscher oder englischer Sprache, die die GK oder UGK im Kontext von Umweltrisiken oder -schadstoffen an einer Population untersuchen. Die Bewertung der methodischen Studienqualität erfolgte mithilfe des Navigation Guide.

**Ergebnisse:**

24 Studien wurden in die qualitative Analyse eingeschlossen. Die Operationalisierung der GK bzw. UGK erfolgte in 22 Studien heterogen. 9 Studien bezogen sich auf Umweltthemen im Allgemeinen und 15 Studien analysierten einzelne Umweltrisiken. 11 Studien bezogen sich auf die allgemeine Bevölkerung, 12 auf spezifizierte Zielgruppen und 1 Studie untersuchte beides. Das Niveau der GK bzw. UGK erwies sich in allen Populationen verbesserungswürdig. Interventionen wie Schulungen konnten die GK bzw. UGK steigern.

**Diskussion:**

Die heterogenen Operationalisierungen erschweren die Vergleichbarkeit der Studien. Zukünftige Arbeiten sollten die Vereinheitlichung methodischer Aspekte forcieren. Insgesamt zeigt sich, dass Interventionen wie Schulungen zur Verbesserung der GK bzw. UGK förderlich waren. Diese sollten in der Praxis zur Erhöhung der GK bzw. UGK verstärkt in den Fokus gerückt werden.

**Zusatzmaterial online:**

Zusätzliche Informationen sind in der Online-Version dieses Artikels (10.1007/s00103-023-03782-5) enthalten. Die zitierten Literaturquellen betreffen die Referenzen [18–42, 48–73].

## Abstract (en)

**Background:**

Environmental factors can play an important role in pathogenesis. However, the understanding of the relationship between environmental risks and health in the general population appears to be poor. The aim of this systematic review is to describe the state of research on the relationship between health literacy (HL) or environmental health literacy (EHL) and environmental risks differentiated by the researched environmental risks, the affected population, the measurement and level of HL or EHL, and their interaction with other variables.

**Methods:**

A systematic literature search was conducted using the Pubmed, Scopus, and LIVIVO databases. Original studies in German or English that investigated HL or EHL in the context of an environmental risk or pollutant in a population were included. The Navigation Guide was used to assess the methodological quality of the studies.

**Results:**

Twenty-four studies were included in the qualitative analysis. The operationalization of HL or EHL was heterogeneous in 22 studies. Nine studies referred to environmental issues in general, and 15 studies analyzed individual environmental risks. Eleven studies referred to the general population, 12 to specified target groups, and 1 study examined both. The level of HL or EHL proved to be in need of improvement in all populations. Interventions such as trainings were able to increase HL or EHL.

**Discussion:**

The heterogeneous operationalizations impede the comparability of the studies. Future work should target the standardization of methodological aspects. Overall, it appears that interventions such as trainings were conducive to improving HL or EHL. In practice, this should therefore be given greater focus in order to increase HL or EHL.

## Hintergrund

Eine „intakte Umwelt“ ist neben ökonomischen und sozialen Faktoren eine wichtige Voraussetzung für ein gesundes Leben [1]. Sie ist dabei ständigen Veränderungen und Einflüssen unterlegen. Vor allem Schadstoffe, die auf sie einwirken, können zu einer Minderung der Umweltqualität führen und die Gesundheit beeinträchtigen [1, 2]. Es gilt bereits als wissenschaftlich gesichert, dass hohe Ozonwerte asthmatische Beschwerden begünstigen können oder an Tagen mit hoher Feinstaubbelastung die Zahl der Krankenhauseinweisungen wegen Herz-Kreislauf-Erkrankungen zunimmt [2]. Der Schutz der Bevölkerung vor diesen Stressoren stellt einen wichtigen Bestandteil einer zukunftsfähigen Entwicklung dar [1], da durch Umweltschutzmaßnahmen ein Beitrag für eine nachhaltige Gesundheitsvorsorge geleistet werden kann [3].

Während Umweltschutz und Umweltbewusstsein in den letzten Jahren zunehmend an Bedeutung gewonnen haben, gestaltet sich das Verständnis der Beziehung zwischen Umweltrisiken und Gesundheit noch schwierig [4, 5]. Um diese Lücke zu schließen, ist es nicht nur erforderlich, Informationen zur Verfügung zu stellen, sondern zu eruieren, inwieweit diese auch verstanden und genutzt werden. Die verschiedenen kognitiven und sozialen Fähigkeiten, die für den Umgang mit und das Verständnis von Gesundheitsinformationen erforderlich sind, werden international als „health literacy“ bezeichnet. Im deutschsprachigen Raum wird der Begriff mit Gesundheitskompetenz (GK) übersetzt [5].

Die GK ist definiert als die Fähigkeit, Gesundheitsinformationen zu finden, zu verstehen, zu bewerten und anzuwenden, um gesundheitsbezogene Entscheidungen treffen zu können. Nutbeam et al. [6] unterteilten sie in 3 Ebenen: funktionell, interaktiv, kritisch. Die funktionelle GK beinhaltet Grundkenntnisse des Lesens und Schreibens sowie die Fähigkeit, diese im Alltag anzuwenden. Die interaktive GK umfasst die Fähigkeit, sich relevante Informationen zu beschaffen, eine Bedeutung abzuleiten und neu erworbenes Wissen auf unterschiedliche Gegebenheiten anzuwenden. Die kritische GK beinhaltet die kritische Analyse von Informationen und die Fähigkeit, diese zu nutzen, um entsprechend zu reagieren, sich anzupassen und Situationen zu kontrollieren [6]. Die GK umfasst somit nicht nur die Fähigkeit zum Lesen oder Schreiben, sondern auch das Wissen, die Motivation und Kompetenzen für eine Meinungsbildung in den Bereichen der Gesundheitsversorgung, Krankheitsprävention und Gesundheitsförderung sowie die Fähigkeit für Entscheidungen, die die Lebensqualität erhalten oder steigern [7–10]. Sie entwickelt sich durch den Einfluss von persönlichen, situativen und gesellschaftlichen Faktoren sowie den Zugewinn an Kompetenz und informelles Lernen im Laufe des Lebens weiter. Dies hat wiederum Auswirkungen auf die Inanspruchnahme des Gesundheitswesens und damit verbundene Kosten, das Gesundheitsverhalten, den Gesundheitszustand, die Partizipation und das Verantwortungsbewusstsein sowie die Nachhaltigkeit und Gerechtigkeit [7].

Eine Untersuchung der GK der erwachsenen Bevölkerung in Deutschland im Jahr 2013 ergab, dass jede dritte Person eine problematische und jede achte Person eine inadäquate GK aufwies, wobei auch soziale Unterschiede innerhalb der Bevölkerungsgruppen sichtbar wurden. Erwachsene mit einem niedrigen Bildungsniveau wiesen eine geringere GK auf als Erwachsene mit höherem Bildungsniveau, was vor allem bei Frauen deutlich ausgeprägt war [5]. Auch der Zusammenhang zwischen gesundheitlichen Auswirkungen und der GK wurde bereits in mehreren Studien festgestellt [6, 11–13].

Ein neuerer Begriff, der nicht nur die Gesundheit allein, sondern auch den Aspekt der Umwelt mit einbezieht, ist die „environmental health literacy“ (umweltbezogene Gesundheitskompetenz [UGK]). In Anlehnung an die GK umfasst sie die Fähigkeiten und Kompetenzen zum Suchen, Verstehen, Bewerten und Anwenden von speziell umweltbezogenen Gesundheitsinformationen, um fundierte Entscheidungen zu treffen, umweltbezogene Gesundheitsrisiken zu verringern, die Lebensqualität zu verbessern und die Umwelt zu schützen [4, 14]. Die UGK beinhaltet Elemente aus verschiedenen Bereichen, wie z. B. GK, Risikokommunikation, umweltbezogene Gesundheit, Kommunikationsforschung und Sicherheitskultur [14].

Der Forschungsbereich zur GK und UGK vergrößert sich ständig, da das Bestreben, die Gesundheit der Menschen zu verbessern, von vielen Fachpersonen aufgegriffen wird [15].

Vor dem Hintergrund der Zunahme der Bedeutung von Umweltrisiken für die Gesundheit scheint eine Untersuchung der GK bzw. UGK in diesem Kontext notwendig. Diese systematische Übersichtsarbeit verfolgt daher das Ziel, den Stand der Forschung zum Zusammenhang zwischen GK bzw. UGK und Umweltrisiken differenziert darzustellen. Dabei werden die untersuchten Umweltrisiken, die Population, die Operationalisierung zur Erhebung und die Ausprägung der GK bzw. UGK sowie Variablen, die einen Zusammenhang mit GK bzw. UGK aufweisen, in den Fokus gerückt.

## Methode

Die systematische Literaturrecherche erfolgte gemäß den Vorgaben des *Cochrane Handbook for Systematic Reviews* und dem *Preferred Reporting Items for Systematic Reviews and Meta-Analyses(PRISMA)-Statement* [16, 17].

Die Suchstrategie wurde auf Basis der beiden Kernaspekte des Forschungsziels entwickelt und setzt sich demnach aus Suchbegriffen zur GK und UGK sowie aus Begriffen im Bereich Umwelt zusammen. Die genauen Suchstrategien in verschiedenen Datenbanken sind in Tab. 1 aufgeführt. Zusätzlich wurde eine Handsuche durchgeführt, um weitere relevante Publikationen oder Querverweise zu finden.

**Table 1:** 

Datenbank	Suchstrategie	Treffer
Pubmed	((″health litera*″[Title/Abstract]) OR (″environmental health litera*″[Title/Abstract])) AND ((″environment*″[Title/Abstract]) OR (″pollut*″[Title/Abstract]) OR (″environmental pollut*″[Title/Abstract] OR ″contamina*″[Title/Abstract] OR ″environmental issue*″[Title/Abstract] OR ″environmental health″[Title/Abstract] OR ″environmental health threat*″[Title/Abstract]))	1193
	(Gesundheitskompetenz) AND (Umwelt* OR Schadstoff* OR Verschmutz* OR Umweltschadstoff* OR Umweltverschmutzung OR Umweltstoff* OR Umweltproblem* OR Umweltgesundheit)	0
Scopus	((″health litera*″ OR ″environmental health litera*″) AND (″environment*″ OR ″pollut*″ OR ″environmental pollut*″ OR ″contamina*″ OR ″environmental issue*″ OR ″environmental health″ OR ″environmental health threat*″))	2137
	ALL (((″gesundheitskompetenz″) AND (″Umwelt*″ OR ″schadstoff* ″ OR ″Verschmutz*″ OR ″umweltschadstoff*″ OR ″Umweltverschmutzung″ OR ″umweltstoff*″ OR ″umweltproblem*″ OR ″Umweltgesundheit″)))	57
LIVIVO	((TI=(health litera* OR environmental health litera*)) OR (KW=(health litera* OR environmental health litera*))) AND ((TI=(″environment*″ OR ″Pollut*″ OR ″environmental pollut*″ OR ″contamin*″ OR ″environmental issue*″ OR ″environmental health″ OR ″environmental health threat*″)) OR (KW=(″environment*″ OR ″Pollut*″ OR ″environmental pollut*″ OR ″contamin*″ OR ″environmental issue*″ OR ″environmental health″ OR ″environmental health threat*″)))	613
	(„Gesundheitskompetenz“) AND („Umwelt*“ OR „Schadstoff*“ OR „Verschmutz*“ OR „Umweltschadstoff*“ OR „Umweltverschmutzung“ OR „Umweltstoff“ OR „Umweltproblem“ OR „Umweltgesundheit“)	27
		∑ 4027

Es wurden Originalarbeiten in deutscher und englischer Sprache eingeschlossen, die die GK oder UGK im Kontext von Umweltrisiken oder -schadstoffen an einer Population untersuchen. Um eine möglichst umfangreiche Analyse der bisher publizierten Literatur vornehmen zu können, wurden keine zeitlichen Beschränkungen vorgenommen (Tab. 2).

**Table 2:** 

	Einschlusskriterien	Ausschlusskriterien
Publikationsart	Originalarbeiten	Bücher, Handbücher, Kongressbeiträge, Kommentare, Letter to the Editor, Dissertationen, Übersichtsarbeiten, Metaanalysen
Endpunkte	Erhebung der GK bzw. UGK in Bezug auf ein Umweltrisiko	Keine Erhebung der GK bzw. UGK in Bezug auf ein Umweltrisiko oder Erhebung in Bezug auf andere Thematiken als Umweltrisiken
Population	Keine Beschränkung auf eine bestimmte Population, aber konkret benannte Population, wie z. B. allgemeine Bevölkerung, definierte Bevölkerungsgruppen, vulnerable Gruppen	Keine Population genannt oder einbezogen
Sprache	Deutsch, Englisch	Andere Sprachen
Veröffentlichungszeitraum	Keine zeitliche Beschränkung	–

*GK* Gesundheitskompetenz, *UGK* Umweltbezogene Gesundheitskompetenz

Die aus den Datenbanken extrahierten Publikationen wurden zunächst um Duplikate bereinigt. Die Prüfung der verbliebenen Studien hinsichtlich der *a priori* definierten Ein- und Ausschlusskriterien wurde von 2 Reviewern unabhängig voneinander vorgenommen. Im Falle von unterschiedlichen Entscheidungen wurde ein dritter Reviewer hinzugezogen.

Für alle eingeschlossenen Studien wurden folgende Daten extrahiert: Autorinnen und Autoren, Jahr, Land, Untersuchung der GK oder UGK, untersuchtes Umweltrisiko, Ziel und Design der Studie, Art der Erhebung, Population, Stichprobengröße, Art der Rekrutierung, Responsezahlen sowie Operationalisierung und Ausprägung der GK bzw. UGK. Zudem wurden Variablen, die mit GK bzw. UGK zusammenhängen, extrahiert. Für die ausgeschlossenen Studien wurde der Grund für den Ausschluss notiert (s. Anhang 1 im Onlinezusatzmaterial).

Die Qualitätsbewertung erfolgte in Anlehnung an Woodruff und Sutton [18] mithilfe des Navigation Guide und umfasst die Aspekte Rekrutierung, Verblindung, Güte der Messinstrumente, Störvariablen, fehlende Werte, Ergebnisbericht, Interessenkonflikt und sonstiger Bias (s. Anhang 2 im Onlinezusatzmaterial).

## Ergebnisse

**Figure 1:**
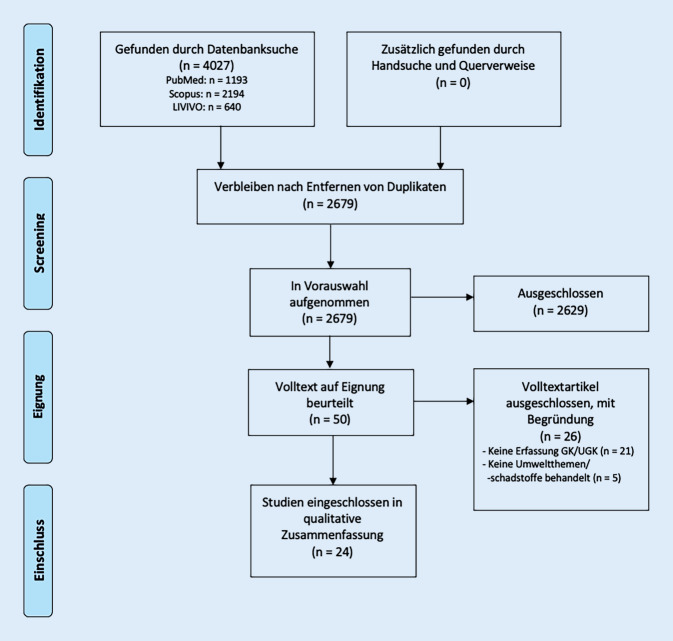

Die Suche erfolgte im November 2022 und lieferte insgesamt 4027 Treffer (Abb. 1). Nach der Bereinigung um Duplikate (*n* = 1348) erfolgte ein Titel- und Abstract-Screening, wobei 2629 Artikel ausgeschlossen wurden. Für die verbliebenen 50 Publikationen wurde ein Volltext-Screening durchgeführt, woraufhin 24 Studien den definierten Einschlusskriterien entsprachen und in die weitere Analyse aufgenommen wurden.

Die eingeschlossenen Studien (Tab. 3) erstrecken sich über den Zeitraum von 13 Jahren (2010–2022). Es wurden 12 Studien in den USA durchgeführt [19–31], 3 im Iran [30, 32, 33], 2 in Italien [34, 35] und jeweils 1 Studie in Taiwan [36], Japan [37], Australien [38], Kenia [39], Ghana [40] und China [41]. Eine Studie machte dazu keine Angabe [42]. Die Hälfte der eingeschlossenen Studien untersuchte die UGK, bei der die Umwelt bereits mit einbezogen ist [20–30, 41], während sich 11 Studien mit der allgemeinen GK im Kontext eines umweltbezogenen Themas befassen [19, 31–38, 40, 42]. Eine Studie untersuchte beide Aspekte [39].

**Table 3:** 

Studie (Land)	GK/UGK (Umweltthema)	Ziel der Studie	Studiendesign, Art der Befragung	Population (Stichprobengröße); Rekrutierung; Antwortrate	Operationalisierung GK/UGK	Ausprägung GK/UGK
Banerjee et al. (2021, [19]; USA)	GK (Sonnenlicht)	Untersuchung der familiären Kommunikation über Hautkrebs bei Patienten in der Primärversorgung, die ganzjährig der Sonne ausgesetzt sind	Querschnittstudie (quantitativ), Fragebogen	Allgemeine Bevölkerung (*n* = 599) Rekrutierung: Register einer Klinik in New Mexico Antwortrate: 99,8 %	Fähigkeit, medizinische Formulare auszufüllen; Häufigkeit von Hilfe beim Lesen von Krankenhausunterlagen; Häufigkeit von Problemen beim Verständnis medizinischer Sachverhalte	Geht nicht daraus hervor – GK wird nur in Zusammenhang mit anderen Variablen untersucht
Binder et al. (2022, [20]; USA)	UGK (PFAS)	Untersuchung der Beziehungen zwischen UGK und soziodemographischen bzw. sozioökonomischen Merkmalen bzgl. der Bereitschaft zu Schutzverhalten	Querschnittstudie (quantitativ), Fragebogen	Allgemeine Bevölkerung (*n* = 726) Rekrutierung: Umfrageplattform Antwortrate: n. a.	Fragen mit wahren oder falschen Fakten über PFAS, zur Wissensadäquanz und zur Handlungskompetenz	Geht nicht daraus hervor – UGK wird nur in Zusammenhang mit anderen Variablen untersucht
Brewer et al. (2019, [21]; USA)	UGK (Umweltthemen allgemein)	Untersuchung der Auswirkung eines Lehrplans zur *Body Balance* auf die UGK	Längsschnittstudie (quantitativ und qualitativ), Fragebogen und Interview	Weiße Frauen > 55 Jahre (*n* = 18–31) Rekrutierung: Radio, Zeitung, Newsletter, Flyer, Facebook-Posts, Mundpropaganda Antwortrate: n. a.	Messung des Wissenszuwachs durch Befragung	Intervention (Lehrplan zu *Body Balance) *hat UGK erhöht
Carducci et al. (2019, [34]; Italien)	GK (Umweltthemen allgemein)	Untersuchung der Wahrnehmung umweltbedingter Gesundheitsrisiken und ihrer Determinanten und Messung der funktionalen GK	Querschnittstudie (quantitativ), Fragebogen	Studierende (*n* = 4778) Rekrutierung: Verteilung von Fragebögen in Vorlesungen Antwortrate: 99 %	Testung des Verständnisses von 12 Begriffen durch Einordnung von Begriffen aus Beipackzetteln in die richtigen Abschnitte eines Textes	55,6 % hohe funktionale GK, 46,4 % geringe funktionale GK
Carducci et al. (2021, [35]; Italien)	GK (Umweltthemen allgemein)	Analyse der Auswirkungen der Wahrnehmung von Gesundheitsrisiken und der funktionalen GK auf umweltfreundliche Einstellungen und Verhaltensweisen	Querschnittstudie (quantitativ), Fragebogen	Studierende (*n* = 4778) Rekrutierung: Anmeldung zur Teilnahme an italienischen Universitäten Antwortrate: 99 %	Testung des Verständnisses von 12 Begriffen durch Einordnung von Begriffen aus Beipackzetteln in die richtigen Abschnitte eines Textes	54 % hohe funktionale GK, 46 % geringe funktionale GK
Davis et al. (2018, [22]; USA)	UGK (Umweltthemen allgemein)	Prä-Post-Befragungen nach UGK-Schulungen in 4 Gemeinden mit bekannten umweltbedingten Gesundheitsrisiken	Querschnittstudie (quantitativ), Fragebogen	Allgemeine Bevölkerung (*n* = 53) Rekrutierung: Informationsstände in der Gemeinde, E‑Mails/Anrufe an Gemeindemitglieder und lokale Organisationen, Pressemitteilungen in lokalen Medien, Anzeigen in Newslettern, Projekt-Harvest-Website Antwortrate: 79 %	Fragen zu umweltwissenschaftlichem Wissen, Fähigkeiten und Motivation für umweltbewusstes Handeln, Selbstwirksamkeit und gemeinschaftlichem Handeln für systemische Veränderungen	UGK durch Intervention (Schulung) signifikant verbessert
Eggers et al. (2018, [23]; USA)	UGK (Wasser)	Bewertung der Gesundheitsrisiken durch verunreinigtes Brunnenwasser in einem Reservat	Querschnittstudie (quantitativ), Fragebogen	Bewohner eines Reservats (*n* = 192) Rekrutierung: Kontakt über Non-Profit-Organisationen Antwortrate: n. a.	Kein konkretes Konstrukt, allgemeine Befragung, von der die UGK abgeleitet wurde	Weitverbreiteter Mangel an UGK
Ghorbani und Heidari (2011, [32]; Iran)	GK (Umweltthemen allgemein)	Untersuchung der Auswirkungen einer Website mit Informationen zur Umwelt auf die GK	Längsschnittstudie (quantitativ), Fragebogen	Schulkinder (*n* = 639) Rekrutierung: Auswahl von Pilotschulen durch das Bildungsministerium Antwortrate: n. a.	Messung des Wissenszuwachses durch Befragung	GK hat sich durch Intervention (Schulung mit Infomaterial auf Website) erhöht mit der geringsten Zunahme bei Umweltgesundheit (+14,5 %) und der größten Zunahme bei Ernährung (+49,9 %)
Gray et al. (2021, [24]; USA)	UGK (Wasser)	Analyse der UGK im Kontext von Wasser mittels eines neuen Tools	Querschnittstudie (quantitativ), Fragebogen	Allgemeine Bevölkerung (*n* = 23), Studierende (*n* = 24) Rekrutierung: E‑Mail und persönliche Treffen mit kommunalen Organisationen Antwortrate: n. a.	Water Environmental Health Literacy (WEHL)	Niedrige GK in der allgemeinen Bevölkerung Angemessene UGK bei Studierenden
Hashemi et al. (2012, [33]; Iran)	GK (Wasser)	Bewertung der GK von Studierenden, um geeignete Bildungsinitiativen vorzuschlagen, mit denen das Wissen erhöht werden kann	Querschnittstudie (quantitativ), Fragebogen	Studierende (*n* = 421) Rekrutierung: n. a. Antwortrate: 100 %	Fragen in Bezug auf das Recycling fester Abfälle	Durchschnittliche GK
Huo et al. (2021, [36]; Taiwan)	GK (Luftschadstoffe)	Untersuchung des Niveaus und der Kovariaten der GK in Zusammenhang mit Luftverschmutzung	Querschnittstudie (quantitativ), Interview	Allgemeine Bevölkerung (*n* = 1297: *n* = 1017 Festnetz, *n* = 280 Mobil); Rekrutierung: über Melderegister Antwortrate: 24,90 % Festnetz, 33,53 % Mobil	Ambient Air Pollution Health Literacy Scale (AAPHL-Scale)	Mäßige GK
Madrigal et al. (2020, [25]; USA)	UGK (Luftschadstoffe)	Entwicklung und Bewertung eines Praktikum-Programms zu den Themen Luftqualität, Gesundheit und kommunale Luftüberwachung für Schulkinder mit dem Ziel, die Gesundheitskompetenz zu erhöhen	Längsschnittstudie (quantitativ), Fragebogen	Schulkinder (*n* = 29) Rekrutierung: Angebot der Teilnahme über Comite Civico del Valle bei Schulkindern, Familien, Gemeindeorganisationen, Schulbezirken und informelle Gespräche mit Gemeindemitgliedern Antwortrate: 100 %	Erfassung der prozentualen Zustimmung zu Aussagen über GK im Umweltbereich	Erhöhung der UGK durch Intervention (Praktikum)
Moriyama et al. (2020, [37]; Japan)	GK (radioaktive Strahlung)	Untersuchung der GK der Einwohner der Präfektur Fukushima nach dem Unfall im Kernkraftwerk	Querschnittstudie (quantitativ), Fragebogen	Bewohner eines Gebiets (Fukushima) (*n* = 770) Rekrutierung: Anschreiben von zufällig ausgewählten Bewohnern aus 4 Teilgebieten Antwortrate: 45,8 % (valid. 38,5 %)	„Communicative and critical health literacy score“ (CCHL-Score)	Niedrige GK
Ragusa und Crampton (2020, [38]; Australien)	GK (Sonnenlicht)	Untersuchung der GK der Befragten in Bezug auf nationale Richtlinien	Querschnittstudie (quantitativ), Fragebogen	Angestellte an Universitäten (*n* = 60) Rekrutierung: Bekanntmachung der Umfrage auf der Online-Kommunikationsplattform der Universität Antwortrate: n. a.	Fragen mit Antwortoptionen, die die aktuellen nationalen Richtlinien und verbreitete Stereotype enthalten	Keine konkrete Erfassung, aber Feststellung, dass das angenommene Wissen zur Sonnenexposition weit entfernt von tatsächlich empfohlenen Richtlinien lag
Ramirez-Andreotta et al. (2016, [26]; USA)	UGK (Arsen)	Bewertung der Lernergebnisse der Teilnehmenden von Umweltkommunikationsmaßnahmen nach einer Biomonitoring-Studie	Querschnittstudie (qualitativ), Interview	Eltern (*n* = 17) Rekrutierung: Teilnehmende einer vorausgegangenen Biomonitoring-Studie Antwortrate: n. a.	Kein konkretes Konstrukt, allgemeine Befragung, aus der die UGK abgeleitet wurde	UGK hat sich erhöht, wenn Eltern Rückmeldung zu den Ergebnissen bekommen haben (Biomonitoring-Studie) und durch Broschüren/Infomaterialien und Gespräche mit Studienleitung
Ramos et al. (2012, [27]; USA)	UGK (Wasser, Luftschadstoffe)	Ermittlung der Auswirkungen einer Schulung auf die UGK	Längsschnittstudie (quantitativ), Fragebogen	Allgemeine Bevölkerung (*n* = 489) Rekrutierung: Zufallsstichprobe anhand einer Karte des Gebiets Antwortrate: 100 %	Kein konkretes Konstrukt, allgemeine Befragung, aus der die UGK abgeleitet wurde	UGK durch Intervention (Schulung) erhöht
Raufman et al. (2020, [39]; Kenia)	GK und UGK (Brennstoffe (in Kochräumen))	Evaluation des Zusammenhangs zwischen UGK und Symptomen durch Brennstoffe in Kochräumen	Querschnittstudie (quantitativ), Fragebogen	Allgemeine Bevölkerung (*n* = 353) Rekrutierung: Anschreiben von zufällig ausgewählten Haushalten Antwortrate: 98 %	Fragen aus früheren validierten, nicht-amerikanischen Erhebungen zur GK und UGK	Geht nicht daraus hervor – GK und UGK werden nur in Zusammenhang mit anderen Variablen untersucht
Simonds et al. (2019, [28]; USA)	UGK (wasserbezogene Umweltkenntnisse)	Durchführung einer Machbarkeitsstudie über ein Schulungsprogramm zur Verbesserung der UGK von Kindern	Längsschnittstudie (quantitativ), Fragebogen	Kinder (*n* = 44) Rekrutierung: Briefe an Eltern, Flyer, Posts in sozialen Medien, Anzeige in lokalem Newsletter Antwortrate: n. a.	Fragen zu wasserbezogenem Grundwissen, zur Fähigkeit, neu erworbene Informationen in sozialen Netzwerken zu teilen, und zum Verhalten und der Einstellung aufgrund des Wissens	UGK hat sich durch Intervention (Schulungsprogramm) erhöht
Stanifer et al. (2022, [29]; USA)	UGK (Radon)	Bewertung der Veränderungen in der UGK und der Wirksamkeit von Schulungen	Längsschnittstudie (quantitativ), Fragebogen	Personen mit Immobilienbesitz (*n* = 60) Rekrutierung: soziale Medien, lokale Zeitungen und Radio, Flyer und Mailinglisten Interessierte Personen: Online-Screening-Umfrage, um Teilnahmeberechtigung zu ermitteln; wenn berechtigt: Kontaktformular Antwortrate: Baseline: 100 % Post-Schulung: 100 % Follow-up: 97 %	Abfrage von Wissen über Radonexposition, die auf der Erhebung der UGK von Finn und O’Fallon 2017 basiert	Intervention (Radonschulung) hat zu signifikanter Erhöhung der UGK geführt
Tavakoly Sany (2022, [30]; Iran)	UGK (Umweltthemen allgemein)	Untersuchung der Konzeptualisierung der UGK durch Eltern	Querschnittsstudie (quantitativ), Interview	Eltern (*n* = 35) Rekrutierung: Anfrage über die Kontaktliste eines Kindergartens Antwortrate: n. a.	Kein konkretes Konstrukt, allgemeine Befragung, aus der die UGK abgeleitet wurde	Mangelhafte UGK in Bezug auf Wissen über Quellen und Auswirkungen toxischer Belastungen, die die Gesundheit der Kinder beeinträchtigen
Tomsho et al. (2022, [31]; USA)	GK (NO_2_, PM_2,5_)	Untersuchung der Wahrnehmung der Luftqualität	Querschnittstudie (qualitativ), Interview	Allgemeine Bevölkerung (*n* = 20) Rekrutierung: Teilnehmende einer vorgelagerten Monitoring-Studie Antwortrate: n. a.	BRIEF-Test (Behavior Rating Inventory of Executive Function)	Geringe bis ausreichende GK
Tutu et al. (2019, [40]; Ghana)	GK (Umweltthemen allgemein (Cholera-Risikofaktoren))	Untersuchung des Grundwissens der Menschen über Cholera-Risikofaktoren	Querschnittstudie (quantitativ), Fragebogen	Bewohner eines Gebiets (Reservat) (*n* = 401) Rekrutierung: Kontakt über Haushaltsverzeichnis Antwortrate: n. a.	Fragen zu Lebensmittelsicherheit und Hygiene	Geht nicht daraus hervor – GK wird nur in Zusammenhang mit anderen Variablen untersucht
Villagran et al. (2010, [42]; n. a.)	GK (allgemeine Umweltthemen (Klimawandel und globale Erwärmung))	Untersuchung der Beziehungen zwischen der GK und Umweltrisiken zur Erstellung von evidenzbasierten Empfehlungen für Gesundheitsdienstleistende zur Aufklärung von Patienten	Querschnittstudie (quantitativ), Fragebogen	Patienten (Consumer Style: *n* = 11.758 Health Style: *n* = 4398 Youth Style: *n* = 1357) Rekrutierung: Teilnehmende vorausgegangener 3‑teiliger Verbraucherumfrage (Consumer Style, Health Style, Youth Style) Antwortrate: Consumer Style: 58,8 % Health Style: 66,6 % Youth Style: 52,8 %	Fragen zum Verständnis von Gesundheit und Krankheit, selbst eingeschätzte Sprech- und Hörfähigkeiten bei Gesundheitsthemen, Bereitschaft zum Lesen und Schreiben über Gesundheitsthemen, Vertrautheit mit gesundheitsbezogenen Daten	Geht nicht daraus hervor – GK wird nur in Zusammenhang mit anderen Variablen untersucht
Zhao et al. (2022, [41]; China)	UGK (allgemeine Umweltthemen)	Analyse von Rahmenbedingungen und Einflussfaktoren für das Niveau der UGK der Bevölkerung und Schaffung einer wissenschaftlichen Grundlage für die Erforschung neuer Ideen und Methoden zur Verbesserung der UGK	Querschnittstudie (quantitativ), Interview	Allgemeine Bevölkerung (*n* = 1320) Rekrutierung: n. a. Antwortrate: 96 %	Abfrage in Anlehnung an das vom chinesischen Umweltministerium entwickelte Konstrukt *The Core Questions for Assessment of Environmental Health Literacy of Chinese Citizens (Trial Implementation)*	Geht nicht daraus hervor – UGK wird nur in Zusammenhang mit anderen Variablen untersucht

*GK* Gesundheitskompetenz, *UGK* Umweltbezogene Gesundheitskompetenz, *n. a.* nicht angegeben, *NO*_*2*_ Stickstoffdioxid, *PM2,5* Feinstaubpartikel mit einem aerodynamischen Durchmesser kleiner als 2,5 µm

**Figure 2:**
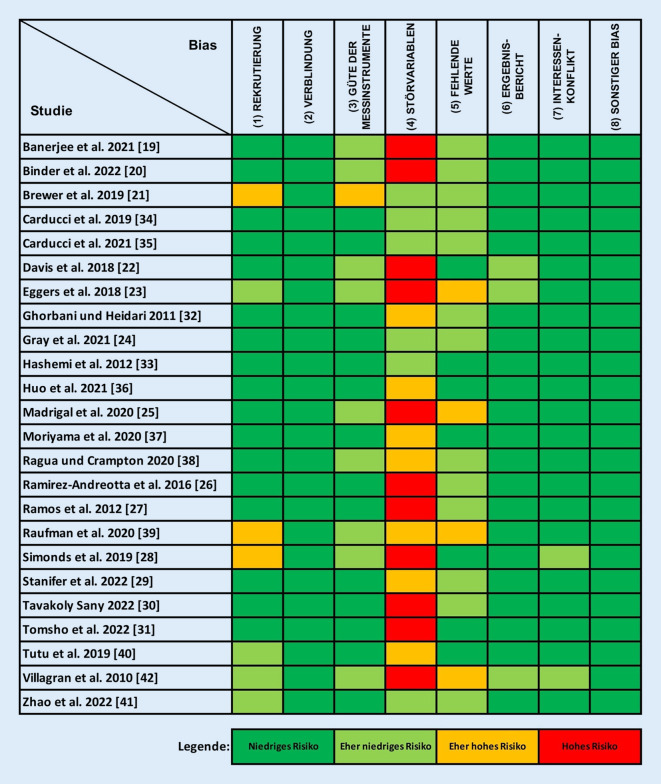

Die Studienqualität hinsichtlich eines Risk of Bias (Abb. 2) ergab für das Kriterium „Störvariablen“ bei 18 der 24 Studien ein „eher hohes“ (7) oder „hohes“ Risiko (11). Ein „eher hohes“ Risiko ergab sich bei 4 Studien für „fehlende Werte“, bei 3 Studien für „Rekrutierung“ und bei 1 Studie für „Güte der Messinstrumente“. Bei den restlichen Kriterien „Verblindung“, „Ergebnisbericht“, „Interessenkonflikt“ und „sonstiger Bias“ wurden für alle Studien „eher niedrige“ oder „niedrige“ Risiken festgestellt.

### Operationalisierung der GK bzw. UGK

Die GK bzw. UGK wurde durch Abfrage der dafür notwendigen Fähigkeiten mittels Fragebögen [19, 20, 22, 24, 25, 27–29, 32–35, 37–40, 42] oder Interviews [23, 26, 30, 31, 36, 41] erfasst. Eine Studie wandte beide Methoden an [21]. Simonds et al. [28] stellten Fragen zu wasserbezogenem Grundwissen, zur Fähigkeit, neu erworbene Informationen in sozialen Netzwerken zu teilen, und zum Verhalten und der Einstellung aufgrund des Wissens. Davis et al. [22] nutzten Fragen aus den Bereichen umweltwissenschaftliches Wissen, Fähigkeiten und Motivation für umweltbewusstes Handeln, Selbstwirksamkeit und gemeinschaftliches Handeln für systemische Veränderungen vor und nach einer Intervention. Binder et al. [20] verwendeten Fragen mit wahren oder falschen Fakten über per- und polyfluorierte Alkylverbindungen (PFAS), Fragen zur Wissensadäquanz (Diskrepanz zwischen angestrebtem und tatsächlichem Wissen) und Fragen zur Handlungskompetenz, d. h., wie wirksam Verhaltensweisen bei der Vermeidung einer Exposition waren. Ein ähnliches Vorgehen ist bei Zhao et al. [41] zu finden, wobei Grundkonzepte (Verständnis der Beziehung zwischen Umwelt und Gesundheit), Grundkenntnisse (Wissen zu Umweltthemen) und Grundfertigkeiten (Verhaltensweisen und Fertigkeiten für den Erwerb, das Verständnis und die Anwendung relevanter Informationen) abgefragt wurden, die auf dem vom chinesischen Umweltministerium entwickelten Konstrukt *The Core Questions for Assessment of EHL of Chinese Citizens (Trial Implementation)* basierten.

2 Studien fokussierten bei der Erfassung der GK bzw. UGK eher medizinische Aspekte [19, 42]. Villagran et al. [42] nutzten Fragen zum Verständnis von Gesundheit und Krankheit, zu den selbst eingeschätzten Sprech- und Hörfähigkeiten in Bezug auf Gesundheitsthemen, zur Bereitschaft zum Lesen und Schreiben über Gesundheitsthemen sowie zur Vertrautheit mit gesundheitsbezogenen Daten [42]. Banerjee et al. [19] bezogen ihre Fragen auf die Fähigkeit, medizinische Formulare auszufüllen, die Häufigkeit von Hilfe beim Lesen medizinischer Daten und von Problemen beim Verständnis medizinischer Sachverhalte. Carducci et al. [34, 35] untersuchten die funktionale GK, mittels Einordnung von Begriffen aus Beipackzetteln in die richtigen Abschnitte eines Textes [34, 35]. Weitere Verfahren waren die Abfrage des Wissens bzw. Wissenszuwachs zu Gesundheitsthemen mittels Punktbewertung [32], die Erfassung der prozentualen Zustimmung zu Aussagen über GK im Umweltbereich [25] sowie Fragen zum Recycling fester Abfälle [33] oder zur Lebensmittelsicherheit und Hygiene [40]. Ragusa und Crampton [38] nutzten Fragen mit Antwortoptionen, die sowohl Aussagen aus offiziellen Leitlinien beinhalteten als auch Optionen, die auf unwahren, in den Medien verbreiteten Stereotypen beruhten. Auch bestehende Tools wurden herangezogen, wie die *Communicative and Critical Health Literacy Scale* [37], der *Behavior Rating Inventory of Executive Function-Test *(BRIEF-Test; [31]), Fragen aus validierten, nicht-amerikanischen Erhebungen zur GK [39] oder auch eine Abfrage von Wissen über Radonexposition, die auf der Erhebung der UGK von Finn und O’Fallon [14] basiert [29].

Zudem wurden 2 neue Tools entwickelt: die *Ambient Air Pollution Health Literacy Scale*, die sich speziell auf GK in Zusammengang mit Luftschadstoffen fokussiert [36], und die *Water Environmental Health Literacy Scale*, die die GK im Kontext von Wasser erfasst [24].

### Umweltthemen

9 Studien bezogen sich auf das Thema Umwelt im Allgemeinen [21, 22, 30, 32, 34, 35, 40–42]. Villagran et al. [42] beschränkten sich dabei auf den Klimawandel und die globale Erwärmung. Tutu et al. [40] nahmen ihre Analysen speziell im Kontext von Cholera vor. Einige Medien und Schadstoffe wurden konkret untersucht, wie z. B. Wasser [23, 24, 27, 28, 33], Luftschadstoffe allgemein [25, 27, 36], PM_2,5_-Feinstaub und Stickstoffdioxid (NO_2_; [31]) sowie Brennstoffe in Kochstätten [39]. Weitere untersuchte Themen waren Sonnenlicht [19, 38], radioaktive Strahlung [37], PFAS [20], Radon [29] und Arsen [26].

### Population

In 9 Studien erfolgte die Untersuchung an der allgemeinen Bevölkerung [19, 20, 22, 24, 27, 31, 36, 39, 41] sowie in 3 Studien mit Bezug auf ein bestimmtes Gebiet [40], wie z. B. Fukushima [37] oder ein Reservat [23]. Speziell untersuchte Zielgruppen waren (Schul‑)Kinder [25, 28, 32], Eltern [26, 30], Studierende [24, 33–35], weiße Frauen über 55 Jahre [21], Angestellte einer Universität [38], Personen mit Immobilienbesitz [29] sowie Patientinnen und Patienten [42].

### Ausprägung der GK bzw. UGK

3 Studien in der allgemeinen Bevölkerung [23, 24, 36] und 1 Studie mit Eltern [30] fanden eine mangelhafte GK. Auch Ragusa et al. [38] stellten fest, dass das angenommene Wissen der Teilnehmenden weit entfernt von empfohlenen Richtlinien lag. Bei Analysen zur GK nach dem Reaktorunfall in Fukushima wurde bei der Bevölkerung der Präfektur eine niedrige GK festgestellt, die unter dem Niveau der Allgemeinbevölkerung lag [37]. Tomsho et al. [31] fanden ein „geringes bis ausreichendes“ Niveau in der allgemeinen Bevölkerung. Bei Untersuchungen mit Studierenden wurde eine „durchschnittliche“ [33] bzw. „angemessene“ GK [24] sowie eine hohe funktionale GK konstatiert [34, 35].

In Studien, die eine Intervention beinhalteten, wurde eine Erhöhung der GK bzw. UGK festgestellt [21, 22, 25–29, 32]. Die Interventionen umfassten ein Praktikum bzw. eine Schulung für Kinder zu umweltbezogenen Aspekten [25, 28], die Erstellung einer Website mit Informationen für Schulkinder [32], Schulungen der allgemeinen Bevölkerung in von Umweltrisiken betroffenen Gemeinden [22, 27], wovon eine Studie zusätzlich die Ausbildung von Trainingspersonen zur Durchführung dieser Schulungen beinhaltete [27], Radonschulungen für Personen mit Immobilienbesitz [29], einen Lehrplan für einen gesunden Lebensstil für weiße Frauen mittleren Alters [21] sowie persönliches Feedback oder Informationsmaterialien für Eltern zu den Ergebnissen einer Biomonitoring-Studie, an der ihre Kinder teilgenommen hatten [26]. Eine Studie stellte fest, dass die Steigerung der GK in Bezug auf Umweltgesundheit (14,5 %) am geringsten und beim Thema Ernährung (49,9 %) am stärksten ausgeprägt war [32].

### Interaktion der GK bzw. UGK mit anderen Variablen

Die GK bzw. UGK wurde auch im Kontext anderer Variablen (Geschlecht [33, 34, 41], Alter [24, 37, 41], Bildung [33, 34, 36, 37, 41], Wohngebiet [33, 34, 36, 41] und -situation [33, 36], Einkommen [33, 41], Kommunikation [19] und Information [34, 42], Risikobewusstsein [31, 34, 42], gesundheitliche Aspekte [39, 40] und (Umwelt‑)Schutzverhalten [20, 35]) untersucht.

In 2 Studien zeigte sich bei Männern ein signifikant höheres Niveau an GK bzw. UGK als bei Frauen [33, 41]. Carducci et al. [34] fanden keinen Unterschied.

2 Untersuchungen stellten für jüngere Personen zwischen 25 und 34 Jahren [41] bzw. unter 24 Jahren [24] eine höhere GK bzw. UGK als bei älteren Befragten fest. Moriyama et al. [37] fanden keinen Zusammenhang der GK mit dem Alter.

Eine höhere GK bzw. UGK wurde mit einem höheren Bildungsniveau assoziiert und umgekehrt [33, 34, 36, 37, 41]. Zudem zeigten die Fachrichtung von Studiengängen und die Semesteranzahl einen Zusammenhang mit der GK. Je mehr Semester absolviert wurden, desto höher war die GK [33, 34]. Die GK war nach Carducci et al. [34] bei Studierenden der Natur- und Lebenswissenschaften geringer als in Human‑, Sozial- und Rechtswissenschaften. Hashemi et al. [33] fanden die höchsten GK-Werte in den Studiengängen Umwelttechnik, Arbeitsmedizin, Ernährungswissenschaften und Public Health. Studierende der Statistik und Epidemiologie wiesen die geringste GK auf. Die Bildung der Eltern zeigte keinen Einfluss auf die GK.

Hou et al. [36] konstatierten, dass Personen mit Kindern < 12 Jahren gegenüber Alleinlebenden sowie Alleinlebende gegenüber Verheirateten eine signifikant höhere GK besaßen. Hashemi et al. [33] fanden bei der Analyse der Anzahl an Familienmitgliedern keinen Zusammenhang mit der GK.

Das Niveau der GK unterschied sich je nach Wohngebiet [33, 34, 36]. Zhao et al. [41] stellten bei Stadtbewohnerinnen und -bewohnern eine signifikant höhere UGK (29,0 %) als bei Menschen vom Land (12,4 %) fest und fanden zudem eine positive Korrelation des Einkommens mit der GK. Hashemi et al. [33] stellten keinen Zusammenhang mit dem Einkommen fest.

Eine höhere GK war in einer Studie signifikant mit einer stärkeren Kommunikation zu Umweltrisiken verbunden [19]. Villagran et al. [42] fanden, dass sich Befragte mit hoher GK gegenüber niedriger GK signifikant „wohler“ fühlten, wenn sie Informationen von Gesundheitsdienstleistenden erhielten. Carducci et al. [34] identifizierten die GK als Prädiktor für das Vertrauen in Institutionen als Informationsquellen, als wichtige Einrichtungen und als wirksame Akteure in Zusammenhang mit umweltbedingten Gesundheitsrisiken. Mehr Vertrauen in das Gesundheits- oder Umweltministerium, regionale Umweltschutzbehörden oder Forschungseinrichtungen und Universitäten bei hoher GK standen dabei einer Tendenz für mehr Vertrauen in soziale Netzwerke und Fachleute für alternative Medizin bei niedriger GK gegenüber. Außerdem wurden die eigenen Kenntnisse zu Risiken bei hoher GK als zufriedenstellender bewertet (31,7 %) als bei niedriger GK (25,8 %). Weiterhin fanden sie, dass Umweltrisiken bei hoher GK häufiger richtig eingeschätzt wurden als bei niedriger GK [34]. Eine weitere Studie stellte fest, dass bei hoher GK die Luftqualität positiver wahrgenommen wurde als bei niedriger GK [31], und Villagran et al. fanden heraus, dass Personen mit hoher GK über mehr Wissen zu Risiken von Umweltschadstoffen verfügten als solche mit niedriger GK [42].

Eine Studie stellte für Personen mit hoher UGK gegenüber Personen mit niedriger UGK ein geringeres Risiko fest, gesundheitliche Symptome zu entwickeln [39], und eine weitere Untersuchung konstatierte bei hoher GK gegenüber niedriger GK eine geringere Wahrscheinlichkeit, ernsthaft zu erkranken [40].

In einer Untersuchung wurde ein positiver Zusammenhang zwischen der GK und der Bereitschaft, Schutzmaßnahmen zu ergreifen, gefunden [20] und eine andere Studie stellte einen positiven Zusammenhang zwischen der Einstellung zu umweltfreundlichem Verhalten und der GK fest [35].

## Diskussion

Das Review behandelt den Zusammenhang zwischen UGK bzw. GK und Umweltrisiken. Hierbei zeigten sich bei der Operationalisierung sehr heterogene Ergebnisse, da diese in nahezu allen Studien unterschiedlich umgesetzt wurde. Nur 2 Studien erfassten diese auf die gleiche Weise [34, 35]. Die durchweg unterschiedliche Operationalisierung erschwert die Vergleichbarkeit der Studien.

Bei den untersuchten Umweltthemen ist festzustellen, dass 14 von 24 Studien einen bestimmten Stoff oder Risikofaktor fokussieren [19, 20, 23–29, 31, 33, 36–39]. Die Spannweite an Umweltfaktoren ist jedoch viel breiter und umfasst auch weitere Stoffe und Faktoren, wie z. B. Ozon, Ultrafeinstaub, Lärm oder Pollen, denen bislang noch keine Aufmerksamkeit in diesem Kontext beigemessen wurde, obwohl auch sie ein Umweltrisiko für die Bevölkerung und vor allem vulnerable Gruppen darstellen können [43, 44]. Vulnerable Gruppen wie Kinder [25, 28, 32] wurden zudem in nur wenigen Studien untersucht. In 9 Studien wurde sich auf die Allgemeinbevölkerung bezogen [19, 20, 22, 24, 27, 31, 36, 39, 41]. Schwangere oder ältere Personen, die ggf. zusätzlich mit Vorerkrankungen belastet sind und deren Gesundheit es in besonderer Weise vor Umweltrisiken zu schützen gilt, wurden nicht betrachtet. Einzig Eltern wurden als Zielgruppe, die in Verbindung mit einer vulnerablen Gruppe steht, in 2 Untersuchungen berücksichtigt [26, 30]. Hierbei wurde zwar ein insgesamt geringes Niveau an GK festgestellt [26, 30], was hinsichtlich der Tatsache, dass sie eine Fürsorgepflicht gegenüber einer schutzbedürftigen Gruppe haben, zu hinterfragen ist. Allerdings zeigte eine weitere Untersuchung, dass Eltern von jüngeren Kindern eine höhere GK aufwiesen als Alleinlebende [36], was darauf hinweist, dass die Fürsorge für diese vulnerable Gruppe dazu beizutragen scheint, sich Kompetenz zum Erhalt deren Gesundheit anzueignen. Zudem gab es vielversprechende Ansätze, z. B. mittels direkten Feedbacks oder Informationsmaterialien, die die GK dieser Population erhöhen und damit zur Gesundheit der Kinder beitragen können [26].

Bei der Erfassung der GK bzw. UGK zeigte sich, dass diese v. a. in der Allgemeinbevölkerung gering und unzureichend ist [23, 24, 31, 36]. Bei Studierenden konnte hingegen ein „durchschnittliches“ bis „hohes“ Niveau an GK konstatiert werden, was damit zu erklären ist, dass sowohl ein höheres Bildungsniveau als auch ein jüngeres Alter mit einer besseren GK bzw. UGK in Verbindung stehen [24, 33]. Diese beiden Aspekte sind in der Population der Studierenden häufig zutreffend. Weiterhin wurde festgestellt, dass die GK bzw. UGK innerhalb der Population der Studierenden unterschiedlich ausgeprägt war [33]. Dies ist möglicherweise auf die fachspezifischen Curricula zurückzuführen – so können z. B. die Lehrinhalte der Studiengänge Umwelttechnik oder Public Health dazu beigetragen haben, dass die GK bzw. UGK dieser Studierenden höher ist als in Studiengängen, deren Schwerpunkt weniger stark auf Umweltthemen liegt, wie z. B. Statistik.

Als geeignete Möglichkeit zur Förderung der GK und UGK erwiesen sich Interventionen wie Schulungen, Praktika oder persönliches Feedback, da durch deren Einbindung stets eine Verbesserung festgestellt wurde [21, 22, 25–29, 32]. Auch der Kommunikation und Information über verschiedene Medien kommt eine Schlüsselrolle zu [19]. Je nach Niveau der GK werden unterschiedliche Informationsquellen bevorzugt. Personen mit niedriger GK griffen häufiger auf soziale Medien zurück, während Personen mit hoher GK eher Informationen institutioneller Einrichtungen präferierten [34, 42]. Da in sozialen Netzwerken Informationen ungefiltert und ohne Überprüfung verbreitet werden können, besteht die Gefahr, dass auch falsche Informationen weitergetragen werden. Dies birgt das Risiko, dass die GK bzw. UGK dadurch nicht nur nicht gefördert, sondern ggf. sogar verschlechtert werden kann. Allerdings ergibt sich gleichzeitig für Kommunikatorinnen und Kommunikatoren das Potenzial, genau diese Kanäle zu nutzen, um wissenschaftlich fundierte Informationen weiterzuverbreiten, die wiederum Zielgruppen erreichen, deren GK bzw. UGK es noch besonders zu fördern gilt.

Einige Studien haben außerdem festgestellt, dass sich eine Erhöhung der GK bzw. UGK positiv auf die Gesundheit auswirkt, da sie dazu befähigt, Umweltrisiken eher zu erkennen [34, 42] und eigenständig Schutzmaßnahmen zu ergreifen [20], sowie die Wahrscheinlichkeit erhöht, nicht bzw. nicht schwer zu erkranken [39, 40].

Zhao et al. [41] stellten einen Unterschied bei der UGK von Stadt- und Landbewohnerinnen und -bewohnern in China fest. Dieser Zusammenhang wurde auch von Aljassim und Ostini [45] untersucht, die zu dem Schluss kamen, dass diese Unterschiede durch einen unterschiedlichen Zugang zur Gesundheitsversorgung je nach Wohngegend erklärt werden können, der für Menschen auf dem Land deutlich schlechter ist [45]. Dies ist auch in China, wo die Studie von Zaho et al. [41] durchgeführt wurde, nach wie vor problematisch [46].

Es fällt auf, dass keine der Studien aus Deutschland stammt. Selbst auf europäischer Ebene sind nur 2 Studien aus Italien zu finden [34, 35]. Obwohl bei der Literaturrecherche keine zeitlichen Einschränkungen vorgenommen wurden, ist zudem festzustellen, dass die Forschung nicht weit in die Vergangenheit zurückreicht. Die erste der Forschungsfrage entsprechende Studie stammt aus dem Jahr 2010. Dies lässt sich darauf zurückführen, dass GK ein noch junger Forschungsbereich ist, der sich erst seit den 1990er-Jahren entwickelt [5]. Da sich die Studien jedoch kontinuierlich bis ins Jahr 2022 fortsetzen, zeigt sich, dass diese Entwicklung weiterhin anhält. Die nicht allzu umfassende Studienlage aufgrund des noch jungen Forschungsbereichs ist auch eine mögliche Erklärung für die derzeit kontroversen bzw. nicht eindeutigen Ergebnisse bei den Variablen Geschlecht [33, 34, 41], Einkommen [33, 41] und Alter [24, 37, 41]. Insgesamt bergen diese Erkenntnisse daher viel Potenzial für weitere Forschung, um die durch das Review identifizierten Forschungslücken zu schließen und mehr Klarheit und Evidenz zu liefern.

## Limitationen

Die Literatursuche erfolgte lediglich in den Datenbanken Pubmed, Scopus und LIVIVO. Bei der Auswahl der Suchbegriffe kann nicht ausgeschlossen werden, dass dadurch weitere relevante Treffer übersehen wurden. Da nur Studien in deutscher und englischer Sprache einbezogen wurden, konnten ggf. weitere Erkenntnisse anderssprachiger Veröffentlichungen nicht berücksichtigt werden. Darüber hinaus erschwerte insbesondere die Heterogenität der Operationalisierung die Vergleichbarkeit der Studienergebnisse. Zukünftige Studien sollten vor allem einen Fokus auf die Vereinheitlichung methodischer Aspekte (einheitliche Operationalisierung) legen.

## Fazit

Das Niveau der GK bzw. UGK scheint insgesamt und in allen untersuchten Populationen noch verbesserungsfähig. Da insbesondere Interventionen, wie beispielsweise Schulungen, eine vielversprechende Möglichkeit bieten, die GK und UGK zu erhöhen, sollte verstärkt auf Maßnahmen und Kampagnen gesetzt werden, die durch Wissensvermittlung auf eine Erhöhung der GK bzw. UGK in der jeweiligen Population abzielen. Dabei ist auch eine verstärkte Fokussierung auf vulnerable Gruppen wie Schwangere, Ältere oder Personen mit Vorerkrankungen unerlässlich. Zudem sollten die Medien oder Kanäle, über die Informationen bezogen oder aktiv kommuniziert werden, in künftigen Forschungen stärker berücksichtigt werden. Speziell sollten soziale Medien in den Fokus genommen werden, über die von institutionellen Einrichtungen wissenschaftlich fundierte und adäquat aufbereitete Informationen verbreitet werden können, wodurch eine Steigerung der GK bzw. UGK, vor allem in Populationen mit bislang eher niedriger GK bzw. UGK, erreicht werden könnte. Die Notwendigkeit der Steigerung der GK bzw. UGK wurde auch kürzlich in der „Stellungnahme zum Referentenentwurf eines Bundes-Klimaanpassungsgesetzes des Bundesministeriums für Umwelt, Naturschutz, nukleare Sicherheit und Verbraucherschutz“ des BKK-Dachverbands [47] aufgegriffen. Hierbei wird die Rolle der gesetzlichen Krankenkassen bei der Förderung der GK bzw. UGK betont, die nicht zuletzt aufgrund ihres Beratungsauftrags geeignete Institutionen darstellen, um entsprechende Maßnahmen zu ergreifen. Im Sinne der Gesundheitsförderung und Prävention trägt die Steigerung der GK und UGK schlussendlich dazu bei, die Gesellschaft dazu zu befähigen, ein Verständnis für Umweltrisiken und deren Reduktion zu entwickeln, um das Risiko der Exposition gegenüber schädlichen Umwelteinflüssen zu minimieren und dadurch die eigene Gesundheit zu schützen. Besonders im deutschsprachigen Raum besteht in diesem Zusammenhang noch weiterer Forschungsbedarf.

### Supplementary Information




