# Supplementary material for: Environmental risks and health literacy: a systematic review
Source: Bundesgesundheitsblatt Gesundheitsforschung Gesundheitsschutz. 2023 Oct 12;67(1):85–98. [Article in German] doi: 10.1007/s00103-023-03782-5 (PMC10776732; doi:10.1007/s00103-023-03782-5)
Supplement: Supplementary file 1 [file 103_2023_3782_MOESM1_ESM.pdf]

Onlinezusatzmaterial zum Beitrag von Pfleger et al. (Bundesgesundheitsblatt Heft 1/2024)

Anhang 1:
Entscheidung nach Volltext-Sichtung mit Begründung bei Ausschluss

Referenz	Entscheidung nach Volltext-Sichtung	Begründung bei Ausschluss
Allen et al. 2019 [48]	Ausschluss	keine Erfassung GK/UGK
Banerjee et al. 2021 [19]	Einschluss	-
Binder et al. 2022 [20]	Einschluss	-
Biswas 2019 [49]	Ausschluss	keine Erfassung GK/UGK
Brenner et al. 2015 [50]	Ausschluss	keine Erfassung GK/UGK
Brewer et al. 2019 [21]	Einschluss	-
Carducci et al. 2019 [34]	Einschluss	-
Carducci et al. 2021 [35]	Einschluss	-
Claudio et al. 2018 [51]	Ausschluss	keine Umweltrisiken
Davis et al. 2018 [22]	Einschluss	-
Dellinger et al. 2019 [52]	Ausschluss	keine Erfassung GK/UGK
Eggers et al. 2018 [23]	Einschluss	-
Friel et al. 2004 [53]	Ausschluss	keine Umweltrisiken
Ghorbani und Heidari 2011 [32]	Einschluss	-
Gray et al. 2021 [24]	Einschluss	-
Hashemi et al. 2012 [33]	Einschluss	-
Hou et al. 2021 [36]	Einschluss	-
Koester et al. 2021 [54]	Ausschluss	keine Erfassung GK/UGK
Kreslake et al. 2016 [55]	Ausschluss	keine Erfassung GK/UGK
Laveaux et al. 2018 [56]	Ausschluss	keine Erfassung GK/UGK
Leach et al. 2022 [57]	Ausschluss	keine Erfassung GK/UGK
Madrigal et al. 2020 [25]	Einschluss	-
Madrigal et al. 2016 [58]	Ausschluss	Keine Umweltrisiken
Manna 1984 [59]	Ausschluss	keine Erfassung GK/UGK
Marsili 2016 [60]	Ausschluss	keine Erfassung GK/UGK
Moriyama et al. 2020 [37]	Einschluss	-
Nassar und Salameh 2021 [61]	Ausschluss	keine Umweltrisiken
Odonkor und Sallar 2020 [62]	Ausschluss	keine Erfassung GK/UGK
Paul et al. 2015 [63]	Ausschluss	keine Erfassung GK/UGK
Ragusa und Crampton 2019 [38]	Einschluss	-
Ramirez-Andreotta et al. 2016 [26]	Einschluss	-
Ramírez et al. 2019 [64]	Ausschluss	keine Erfassung GK/UGK
Ramos et al. 2012 [27]	Einschluss	-
Rathinam et al. 2021 [65]	Ausschluss	keine Erfassung GK/UGK
Raufman et al. 2020 [39]	Einschluss	-
Rogers et al. 2014 [66]	Ausschluss	keine Erfassung GK/UGK
Sahoo et al. [67]	Ausschluss	keine Erfassung GK/UGK
Sampson et al. 2021 [68]	Ausschluss	keine Erfassung GK/UGK
Shri und Tiwari 2021 [69]	Ausschluss	keine Erfassung GK/UGK
Simonds et al. 2019 [28]	Einschluss	-
Souto-Miranda et al. 2020 [70]	Ausschluss	keine Erfassung GK/UGK
Stanifer et al. 2022 [29]	Einschluss	-

Tavakoly Sany 2022 [30]	Einschluss	-
Tomsho et al. 2022 [31]	Einschluss	-
Tutu et al. 2019 [40]	Einschluss	-
Vandiver et al. 2022 [71]	Ausschluss	keine Erfassung GK/UGK
Villagran et al. 2010 [42]	Einschluss	-
White et al. 2014 [72]	Ausschluss	keine Erfassung GK/UGK
Yumiya et al. 2020 [73]	Ausschluss	Keine Umweltrisiken
Zhao et al. 2022 [41]	Einschluss	-

Die im Onlinezusatzmaterial angegebenen Literaturreferenzen finden sich im Literaturverzeichnis des Hauptartikels.

Anhang 2:
Bewertungskriterien Risk-of-Bias

Die Bewertung der Studienqualität erfolgte in Anlehnung an Woodruff und Sutton 2014 [18] mithilfe des Navigation Guides und umfasst: Rekrutierung, Verblindung, Güte der Messinstrumente, Störvariablen, fehlende Werte, Ergebnisbericht, Interessenkonflikt und sonstige Bias.
Die Kriterien der einzelnen Aspekte für eine Bewertung als „niedriges Risiko“, „eher niedriges Risiko“, „eher hohes Risiko“, „hohes Risiko“ oder „nicht anwendbar“ sind nachstehend beschrieben:

(1) REKRUTIERUNG:

**Niedriges Risiko**	○ Rekrutierung der Studienteilnehmenden erfolgte zu **einem Zeitpunkt** und aus **einer Population** ODER<br>○ aus **verschiedenen Populationen**, aber der **Anteil** in den jeweiligen Studiengruppen ist **gleich** verteilt
**Eher niedriges Risiko**	Die Rekrutierung wurde **nicht ausreichend beschrieben**, um für ein niedriges Risiko eingestuft zu werden, aber es gibt **Hinweise**, dass die Rekrutierung entsprechend der Kriterien **für ein „niedriges Risiko“** erfolgte.
**Eher hohes Risiko**	Rekrutierung wurde **nicht ausreichend beschrieben**, um für ein hohes Risiko eingestuft zu werden, aber es gibt **Hinweise** darauf, dass die Rekrutierung der Teilnehmenden entsprechend der Kriterien **für ein „hohes Risiko“** erfolgte.
**Hohes Risiko**	○ Das **Vorgehen bei der Rekrutierung** oder die Anwendung der definierten **Ein- und Ausschlusskriterien** erfolgte in den einzelnen Studiengruppen **unterschiedlich** ODER<br>○ Rekrutierung der Studienteilnehmenden erfolgte zu **unterschiedlichen Zeitpunkten** ODER<br>○ aus **verschiedenen Populationen** und der **Anteil** in den jeweiligen Studiengruppen ist **nicht gleich** verteilt ODER<br>○ **unterschiedliche Ausscheideraten** bis zum nächsten Erhebungszeitpunkt<br>○ **Non-Responsezahl** ist zwischen den Gruppen **gleich**
**Nicht anwendbar**	Es gibt Hinweise, dass die Rekrutierung der Teilnehmenden kein Element des Studiendesigns ist, das ein Risk-of-Bias mit sich bringt.

(2) VERBLINDUNG:

**Niedriges Risiko**	○ **Keine Verblindung**, aber Einschätzung der Reviewer, dass das **Ergebnis wahrscheinlich nicht** durch fehlende Verblindung **beeinflusst** wird ODER<br>○ **Verblindung der Studienleitenden** ist gewährleistet, und es ist unwahrscheinlich, dass die Verblindung hätte umgangen werden können ODER<br>○ **die Studienleitenden** waren **nicht verblindet**, aber die **Bewertung der Ergebnisse war verblindet**, und es ist unwahrscheinlich, dass die Nicht-Verblindung anderer Personen zu einer Verzerrung führt
**Eher niedriges Risiko**	Verblindung wurde **nicht ausreichend beschrieben**, um für ein niedriges Risiko eingestuft zu werden, aber es gibt **Hinweise**, dass die Verblindung entsprechend der Kriterien für ein **„niedriges Risiko“** erfolgte.
**Eher hohes Risiko**	Verblindung wurde **nicht ausreichend beschrieben**, um für ein hohes Risiko eingestuft zu werden, aber es gibt **Hinweise** darauf, dass die Studie nicht

	angemessen verblindet war und entsprechend der Kriterien für ein **„hohes Risiko"** erfolgte.
**Hohes Risiko**	○ **Keine Verblindung oder unvollständige Verblindung** und das **Ergebnis** wird wahrscheinlich durch die fehlende Verblindung **beeinflusst** <u>ODER</u><br>○ **Verblindung der Studienleitenden** wurde **angestrebt**, aber es ist wahrscheinlich, dass die Verblindung **umgangen** wurde, so dass es zu einer Verzerrung gekommen sein könnte <u>ODER</u><br>○ **einige der Studienleitenden** waren **nicht verblindet**, und die Nicht-Verblindung anderer Personen hätte wahrscheinlich zu einer Verzerrung führen können.
**Nicht anwendbar**	Es gibt Hinweise, dass die Verblindung der Teilnehmenden kein Element des Studiendesigns ist, das ein Risk-of-Bias mit sich bringt.

(3) GÜTE DER MESSINSTRUMENTE
Da die Forschungsfrage anders als bei Woodruff und Sutton 2014 keine Exposition beinhaltet, wurde sich im gemeinschaftlichen Diskurs dafür entschieden, stattdessen die Güte der Messinstrumente zu bewerten, mithilfe derer die Gesundheitskompetenz bzw. die umweltbezogene Gesundheitskompetenz erhoben wurden.

**Niedriges Risiko**	○ Einsatz von **normierten** oder **validierten Messinstrumenten** <u>ODER</u><br>○ Beschreibung einer **akkuraten Testung** von **neu** entwickelten **Messinstrumenten** (z. B. mindestens zwei Stichproben und Durchführung konfirmatorischer Faktorenanalysen bzw. Strukturgleichungsmodelle)
**Eher niedriges Risiko**	Güte der Messinstrumente wurde **nicht ausreichend beschrieben**, um für ein niedriges Risiko eingestuft zu werden, aber es gibt **Hinweise**, dass die Güte der Messinstrumente den Kriterien für ein **„niedriges Risiko"** entspricht.
**Eher hohes Risiko**	Güte der Messinstrumente wurde **nicht ausreichend beschrieben**, um für ein hohes Risiko eingestuft zu werden, aber es gibt **Hinweise** darauf, dass die Güte der Messinstrumente den Kriterien für ein **„hohes Risiko"** entspricht.
**Hohes Risiko**	○ **Keine Beschreibung** der Güte der verwendeten **Messinstrumente** <u>ODER</u><br>○ die verwendeten **Messinstrumente** sind **nicht normiert** oder **validiert** <u>ODER</u><br>○ es wurden **Messinstrumente neu entwickelt** und es erfolgte **keine Beschreibung** einer **akkuraten Testung**
**Nicht anwendbar**	Es gibt Hinweise, dass die Güte der Messinstrumente kein Element des Studiendesigns ist, das ein Risk-of-Bias mit sich bringt.

(4) STÖRVARIABLEN:
Vor der Bewertung der Studien erfolgte eine gemeinschaftliche Auswahl potenzieller Störfaktoren durch die Autorinnen EP und RL, die auf Basis einer Vorabrecherche in der Literatur ausgewählt wurden:

- **Geschlecht**
- **Alter**
- **Bildung/Einkommen (sozioökonomischer Status)**
- **Gesundheitsstatus**

**Niedriges Risiko**	○ **Alle 4** wichtigen potenziellen Störvariablen wurden berücksichtigt (z. B. gematcht, geschichtet, multivariate Analyse oder anderweitig statistisch kontrolliert) <u>ODER</u><br>○ es wurde **berichtet**, dass potenzielle Störvariablen evaluiert und ausgelassen wurden, weil ihre Einbeziehung die Ergebnisse nicht wesentlich beeinflusst hat.

Eher niedriges Risiko	**3** wichtige potenzielle Störvariablen wurden berücksichtigt UND es ist **nicht zu erwarten**, dass eine fehlende Berücksichtigung die **Ergebnisse wesentlich beeinflusst** hat.
Eher hohes Risiko	**Mind. 2** wichtige potenzielle Störvariablen wurden berücksichtigt, andere potenzielle Störvariablen wurden miteingeschlossen UND die fehlende Berücksichtigung kann zu einer **erheblichen Verzerrung** geführt haben.
Hohes Risiko	**1 oder keine** der wichtigen potenziellen Störvariablen wurden berücksichtigt.

(5) FEHLENDE WERTE:

Niedriges Risiko	o **Keine fehlenden Werte** ODER<br>o Nennung von **Gründen für fehlende Werte**, bei denen es unwahrscheinlich ist, dass sie mit dem tatsächlichen Ergebnis zusammenhängen ODER<br>o **Berichten von Abbrüchen oder fehlenden Werten**, die zahlenmäßig zwischen den Studiengruppen **ausgeglichen** sind, mit ähnlichen Gründen für fehlenden Werte in allen Gruppen ODER<br>o bei dichotomen Daten ist der Anteil der fehlenden Werte im Vergleich zum beobachteten Risiko nicht groß genug, um einen relevanten Einfluss auf die Schätzung des Interventionseffekts zu haben ODER<br>o bei kontinuierlichen Daten reicht die Effektgröße (Mittelwertdifferenz oder standardisierte Mittelwertdifferenz) bei den fehlenden Werten nicht aus, um eine relevante Auswirkung auf die Effektgröße zu haben ODER<br>o **Fehlende Werte** wurden mit geeigneten Methoden **imputiert**
Eher niedriges Risiko	Fehlende Werte wurden **nicht ausreichend beschrieben**, um für ein niedriges Risiko eingestuft zu werden, aber es gibt **Hinweise**, dass diese Werte entsprechend den Kriterien für ein **„niedriges Risiko"** angemessen berücksichtigt wurden.
Eher hohes Risiko	Fehlende Werte wurden **nicht ausreichend beschrieben**, um für ein hohes Risiko eingestuft zu werden, aber es gibt **Hinweise**, dass diese Werte entsprechend den Kriterien für ein **„hohes Risiko"** nicht angemessen berücksichtigt wurden.
Hohes Risiko	o Nennung von **Gründen für fehlende Werte**, die wahrscheinlich mit dem tatsächlichen Ergebnis zusammenhängen, wobei entweder die Anzahl oder die Gründe für fehlende Werte zwischen den Studiengruppen unausgewogen sind ODER<br>o bei dichotomen Daten ist der Anteil der fehlenden Werte im Vergleich zum beobachteten Risiko groß genug, um einen relevanten Einfluss auf die Schätzung des Interventionseffekts zu haben ODER<br>o bei kontinuierlichen Daten reicht die Effektgröße (Mittelwertdifferenz oder standardisierte Mittelwertdifferenz) bei den fehlenden Werten aus, um eine relevante Auswirkung auf die Effektgröße zu haben ODER<br>o **Fehlende Werte** wurden nicht **imputiert**
Nicht anwendbar	Es gibt Hinweise, dass fehlende Werte kein Element des Studiendesigns sind, das ein Risk-of-Bias mit sich bringt.

(6) ERGEBNISBERICHT:

Niedriges Risiko	**Alle** vorgegebenen Ergebnisse der Studie, die im Protokoll, in der Einleitung, den Methoden und/oder der Zusammenfassung beschrieben sind und die für die Überprüfung von Interesse sind, wurden adäquat **angegeben.**
Eher niedriges Risiko	Die Auswahl der berichteten Ergebnisse wurde **nicht ausreichend beschrieben**, um für ein niedriges Risiko eingestuft zu werden, aber es gibt **Hinweise**, dass der Bericht der Ergebnisse gemäß den Kriterien für ein niedriges Risiko **erfolgte.**

**Eher hohes Risiko**	Die Auswahl der berichteten Ergebnisse wurde **nicht ausreichend beschrieben**, um für ein hohes Risiko eingestuft zu werden, aber es gibt **Hinweise**, dass der Bericht der Ergebnisse gemäß den Kriterien für ein hohes Risiko **erfolgte.**
**Hohes Risiko**	○ **Nicht alle** vorgesehenen Ergebnisse der Studie, die im Protokoll, in der Einleitung, den Methoden und/oder der Zusammenfassung beschrieben sind, wurden adäquat **angegeben** ODER<br>○ ein oder mehrere **Ergebnisse** wurden **nicht vorab spezifiziert** (Ausnahme: eine eindeutige Begründung für ihrer Angabe wird genannt, z. B. ein unerwarteter Effekt) ODER<br>○ **Ergebnisse** von Interesse werden **unvollständig** angegeben
**Nicht anwendbar**	Es gibt Hinweise, dass der Ergebnisbericht kein Element des Studiendesigns ist, das ein Risk-of-Bias mit sich bringt.

(7) INTERESSENKONFLIKT:

**Niedriges Risiko**	○ **Kein Interessenkonflikt** ODER<br>○ **keine finanzielle Unterstützung** eines Unternehmens, einer Studienautorin bzw. eines Studienautors oder einer anderen Einrichtung, die ein finanzielles Interesse am Ergebnis der Studie hat
**Eher niedriges Risiko**	Interessenkonflikt oder finanzielle Unterstützung wurden **nicht ausreichend beschrieben**, um für ein niedriges Risiko eingestuft zu werden, aber es gibt **Hinweise** darauf, dass die Studie den Kriterien für ein niedriges Risiko entspricht.
**Eher hohes Risiko**	Interessenkonflikt oder finanzielle Unterstützung wurden **nicht ausreichend beschrieben**, um für ein hohes Risiko eingestuft zu werden, aber es gibt **Hinweise** darauf, dass die Studie den Kriterien für ein hohes Risiko entspricht.
**Hohes Risiko**	○ **Interessenkonflikt** ODER<br>○ **finanzielle Unterstützung** eines Unternehmens, einer Studienautorin bzw. eines Studienautors oder einer anderen Einrichtung, die ein finanzielles Interesse am Ergebnis der Studie hat
**Nicht anwendbar**	Es gibt Hinweise, dass der Interessenkonflikt kein Element des Studiendesigns ist, das ein Risk-of-Bias mit sich bringt.

(8) SONSTIGER BIAS:

**Niedriges Risiko**	**Kein** sonstiger Bias erkennbar.
**Eher niedriges Risiko**	Weiterer Bias wurde **nicht ausreichend beschrieben**, um für ein niedriges Risiko eingestuft zu werden, aber es gibt **Hinweise** darauf, dass die Studie den Kriterien für ein niedriges Risiko entspricht.
**Eher hohes Risiko**	Weitere Bias wurde **nicht ausreichend beschrieben**, um für ein hohes Risiko eingestuft zu werden, aber es gibt **Hinweise** darauf, dass die Studie den Kriterien für ein hohes Risiko entspricht.
**Hohes Risiko**	Es besteht **mindestens ein weiterer Bias** und zwar: __________
